# Psychometric properties of the St. Elizabeth Youngstown hospital wellbeing inventory and non-burnout inventory for physicians and nurses

**DOI:** 10.1186/s40359-019-0316-x

**Published:** 2019-06-17

**Authors:** C. Michael Dunham, Amanda L. Burger, Barbara M. Hileman, Elisha A. Chance

**Affiliations:** 1 Trauma, Critical Care, and General Surgery Services, St. Elizabeth Youngstown Hospital, 1044 Belmont Ave, Youngstown, OH 44501 USA; 2 Behavioral Medicine, St. Elizabeth Family Medicine Residency, 1053 Belmont Ave, Youngstown, OH 44504 USA; 3 Trauma and Neuroscience Research Department, St. Elizabeth Youngstown Hospital, 1044 Belmont Ave, Youngstown, OH 44501 USA

**Keywords:** Stress, Affect, Burnout, professional, Scale validation, Psychometric testing, Physicians, Nurses

## Abstract

**Background:**

Physicians and nurses have substantial problems with wellbeing and burnout. We examined the reliability and construct validity of a wellbeing inventory (WBI) administered to some physicians and nurses working in St. Elizabeth Youngstown Hospital (SEYH).

**Methods:**

The SEYH-WBI, consisting of 4 positive affect (PA) items and 7 negative affect (NA) items developed from 5 validated surveys, was administered (*n* = 419). A non-burnout inventory (SEYH-NBI) consisting of 2 PA items and 3 NA items was derived from the SEYH-WBI. The Positive and Negative Affect Schedule (PANAS), a validated survey consisting of 10 PA items and 10 NA items, was conducted (*n* = 191). The Maslach Burnout Inventory (MBI), a validated survey consisting of 3 domains (3 items each), was completed (*n* = 150).

**Results:**

For the SEYH-WBI, Cronbach coefficients were 0.76 for PA items and 0.83 for NA items. The NA item loading on factor 1 was 0.55–0.84 and the PA item loading on factor 2 was 0.47–0.89. Confirmatory indices were as follows: root mean square residual, 0.07 and Bentler Comparative Fit Index, 0.92. For the SEYH-NBI, Cronbach coefficients were 0.76 for PA items and 0.79 for NA items. The NA item loading on factor 1 was 0.80–0.87 and the PA item loading on factor 2 was 0.89–0.90. Confirmatory indices were as follows: root mean square residual, 0.02; and Bentler Comparative Fit Index, 0.99. PANAS correlations were as follows: SEYH-WBI PA and PANAS PA scores, *r* = 0.9; *p* <  0.0001; SEYH-WBI NA and PANAS NA scores, *r* = 0.9; *p* <  0.0001; SEYH-NBI PA and PANAS PA scores, *r* = 0.8; *p* <  0.0001; and SEYH-NBI NA and PANAS NA scores, *r* = 0.7; *p* <  0.0001. Correlations for SEYH-NBI and MBI were as follows: total NBI and total MBI, *r* = − 0.6, *p* <  0.0001; NA and emotional exhaustion, *r* = 0.6, *p* <  0.0001; PA and personal accomplishment, *r* = 0.3, *p* = 0.0003; and NA and depersonalization, *r* = 0.3, *p* = 0.0008.

**Conclusions:**

Validation assessments indicate that the SEYH-WBI and SEYH-NBI have acceptable psychometric performance. Similar findings in a larger cohort would be more compelling.

## Background

Evidence indicates that physicians and nurses have substantial problems with wellbeing and burnout [1–5]. Apropos, the current authors, in 2017, initiated a study to assess the role of Bispectral Index™ (BIS)-neurofeedback in physician and nurse wellbeing at St. Elizabeth Youngstown Hospital [6]. A survey tool, consisting of 3 positive affect and 7 negative affect items, was created by selecting components from 5 tools in order to follow changes in subject wellbeing [7–11]. Based on results from that pilot study, one new positive affect item was added to the survey tool and a new study was implemented in 2018. That study has been completed and included 57 nurse/physician subjects who underwent 4 neurofeedback learning sessions each (publication pending). Because the wellbeing survey tool was administered on each learning day, 228 surveys were completed. The 11-item survey developed by the current authors will be referred to as the St. Elizabeth Youngstown Hospital Wellbeing Inventory (Wellbeing Inventory).

Our principal intent was to use a wellbeing measure that assessed positive and negative affect constructs in nurses and physicians working in a United States hospital. More specifically, our objective was to assess wellbeing in our BIS neurofeedback study where learning sessions and wellbeing evaluations were occurring weekly for four sessions [6]. That is, the BIS neurofeedback investigation required a concise and focused wellbeing instrument with adequate psychometric performance that would measure wellbeing changes over a relatively short period of time. We also felt that a wellbeing measure with only a modest number of items would facilitate capturing more accurate subject self-appraisals, when compared to a larger set of items. We further sought to determine if an abbreviated or shortened version positive and negative affect construct would correlate with Positive and Negative Affect Schedule (PANAS) and Maslach Burnout Inventory (Maslach) [11–13].

Previous studies have assessed nurse or physician wellbeing, using Maslach and a myriad of other surveys, as it relates to workload, work environment, job satisfaction, patient satisfaction, and coping skills [1–5, 14]. However, we have found no investigations focusing on nurses’ and physicians’ overall wellbeing as it relates to the individual person. The Maslach item responses were problematic, because they include options for time periods much greater than a few days; i.e.; weekly, monthly, few times a year, and never [11]. The Perceived Stress Scale was designed to measure stress over the last month, and this was not suitable for the same reasons that the Maslach was not appropriate [8]. The Depression Anxiety Stress Scales-21 was considered; however, it only measures negative affect [7]. Since restful sleep is essential to overall wellbeing, the Medical Outcomes Study Sleep Scale was considered but was not utilized because it strictly assesses sleep without including any other wellbeing elements [10]. The PANAS was more desirable because it measures the intensity of affects over a week’s time [9]. However, some items of the PANAS seemed redundant (“scared” and “afraid” or “guilty” and “ashamed”). Of importance, the authors’ study design [6] required participants to complete a wellbeing survey roughly every 4–7 days – potentially closer intervals than the weekly period measured in PANAS.

A further problem with commonly used survey tools is the lack of validation in the relevant cohort, physicians and nurses working in a United States hospital. Although PANAS has been validated in several settings, we could find no evidence that PANAS has been validated in a cohort of physicians or nurses working in a United States hospital. Therefore, we did not have any compelling motivation to use the PANAS measure. We found one publication assessing a positive affect and negative affect tool in United States hospital-based nurses and the manuscript provided evidence that the tool had an acceptable Cronbach alpha and exploratory factor analysis results [14]. Because the manuscript did not provide confirmatory factor analysis or concurrent validity results, there was no evidence that the tool had been validated. We could also find no evidence that the Maslach has been validated in physicians or nurses working in a United States hospital.

In addition to the BIS-neurofeedback participants, we administered the Wellbeing Inventory survey in other St. Elizabeth Youngstown Hospital nurses and physicians who concomitantly completed a PANAS survey [9, 15]. From the 11-item Wellbeing Inventory, 5 items were selected to potentially represent indicators for burnout and will be referred to as the St. Elizabeth Youngstown Hospital Non-burnout Inventory (Non-burnout Inventory). A cohort of St. Elizabeth Youngstown Hospital physicians and nurses simultaneously completed the Non-burnout Inventory and the abbreviated Maslach.

On the basis of other literature, we hypothesized that the Wellbeing Inventory would suggest the presence of concerns in physicians and nurses as they relate to the positive affect, negative affect, and burnout results. Further, we hypothesized that the Wellbeing Inventory and Non-burnout Inventory would have adequate validity according to factor analyses. We also hypothesized that the Wellbeing Inventory and Non-burnout Inventory would have concurrent validity with the other relevant and validated survey tools. Therefore, this study examined the reliability and construct validity of a wellbeing inventory administered to a group of physicians and nurses working in St. Elizabeth Youngstown Hospital. Our aspiration was to develop a brief tool with adequate psychometric performance that would measure wellbeing changes over a relatively short period of time.

## Methods

### Ethics approval and subjects

The institutional review board approved the 11-item Wellbeing Inventory that was completed by BIS-neurofeedback participants and required signed, informed consent (number: 17–006; June 20, 2018). The institutional review board approved the concomitant completion of the 11-item Wellbeing Inventory and the 20-item PANAS survey and waived the need for consent (number: 18–031; September 27, 2018). The institutional review board approved the concomitant completion of the 5-item Non-burnout Inventory and the 9-item Maslach and waived the need for consent (number: 18–032; October 11, 2018). The completion of the concomitant surveys posed less than minimal risk and the return of a these questionnaires was interpreted as informed consent.

The present study investigated the reliability and construct validity of a wellbeing inventory administered to physicians or nurses working in St. Elizabeth Youngstown Hospital. Analyses of the 11-item Wellbeing Inventory include internal consistency assessment, exploratory factor analysis, confirmatory factor analysis, and concurrent validity correlations with PANAS. Statistical interrogations of the 5-item Non-burnout Inventory include internal consistency assessment, exploratory factor analysis, confirmatory factor analysis, and concurrent validity correlations with PANAS and with Maslach.

### Wellbeing inventory survey

For the Wellbeing Inventory, the negative affect items included irritation, nervousness, overreaction, tension, overwhelmed, people too demanding, and drained. The positive affect items included restful sleep, energetic, alert, and enthusiastic. The negative affect and positive affect items were rated as very slightly or none at all, a little, moderately, quite a bit, or extremely according to subjects’ experience over the previous 3 days. The Wellbeing Inventory survey was completed by physicians and nurses who participated in the BIS-neurofeedback study and other physicians and nurses who concomitantly completed Wellbeing Inventory and PANAS surveys.

### PANAS survey

For the PANAS, the negative affect items included afraid, ashamed, distressed, guilty, hostile, irritated, jittery, nervous, scared, and upset. The positive affect items incorporated alert, active, attentive, determined, enthusiastic, excited, inspired, interested, proud, and strong. The negative affect and positive affect items were rated as very slightly or none at all, a little, moderately, quite a bit, or extremely according to subjects’ experience over the previous 3 days. The PANAS survey was completed by physicians and nurses who concomitantly completed Wellbeing Inventory and PANAS surveys.

### Maslach survey

The abbreviated Maslach emotional exhaustion domain consisted of the following items: 1) I feel emotionally drained from my work; 2) I feel fatigued when I get up in the morning and have to face another day on the job; and 3) working with people all day is really a strain for me. These items were referred to as drained, fatigued, and strained, respectively. The abbreviated Maslach depersonalization component consisted of the following items: 1) I feel I treat some patients as if they were impersonal objects; 2) I have become more callous toward people since I took this job; and 3) I do not really care what happens to some patients. These items were referred to as objects, callous, and do not care, respectively. The abbreviated Maslach personal accomplishment domain consisted of the following items: 1) I deal very effectively with the problems of my patients; 2) I feel I am positively influencing other people’s lives through my work; and 3) I feel exhilarated after working closely with my patients. These items were referred to as effective, positive influence, and exhilarated, respectively. All items were rated as never, few times a year, once a month or less, few times a month, once a week, a few times a week, or every day. The Maslach survey was completed by physicians and nurses who concomitantly completed Non-burnout Inventory and Maslach surveys.

### Non-burnout inventory surveys

For the Non-burnout Inventory, the negative affect items included overwhelmed, people too demanding, and drained. The positive affect items included energetic and enthusiastic. The negative affect and positive affect items were rated as very slightly or none at all, a little, moderately, quite a bit, or extremely according to participants’ experience over the previous 3 days.

### Factor analyses

A Cronbach alpha coefficient and exploratory and confirmatory factor analyses were conducted to assess the Wellbeing Inventory and Non-burnout Inventory items. For the Wellbeing Inventory confirmatory factor analysis, the 4 positive affect items were coded as 1 for very little or not at all up to 5 for extremely and the 7 negative affect (nonstress) items were coded as 5 for very little or not at all to 1 for extremely. Structural equation modeling was used to model negative affect items and errors as subcomponents of factor 1. Structural equation modeling was used to model positive affect items and errors as subcomponents of Factor 2. The model was composed such that Factor 1 was not related to factor 2.

For the Non-burnout Inventory confirmatory factor analysis, the 2 positive affect items were coded as 1 for very little or not at all up to 5 for extremely and the 3 negative affect items were coded as 5 for very little or not at all to 1 for extremely. Structural equation modeling was used to model negative affect items and errors as subcomponents of factor 1. Structural equation modeling was used to model positive affect items and errors as subcomponents of factor 2. The model was formulated such that factor 1 was not related to factor 2.

### Concurrent validity of the PANAS

Correlation coefficient analyses between PANAS and 11-item Wellbeing Inventory negative affect and positive affect scores were performed. The Wellbeing Inventory positive affect score was the sum of the alert, enthusiastic, energetic, and restful sleep scores. The PANAS positive affect score was the sum of the alert, enthusiastic, interested, excited, strong, proud, inspired, attentive, active, and determined scores. The Wellbeing Inventory negative affect score was the sum of the irritated, nervous, overreaction, tension, overwhelmed, people demanding, and drained scores. The PANAS negative affect score was the sum of the irritated, nervous, distressed, upset, scared, guilty, hostile, ashamed, jittery, and afraid scores. The PANAS and Wellbeing Inventory positive affect items were coded as 1 for very slightly up to 5 for extremely. The PANAS and Wellbeing Inventory negative affect items were coded as 1 for very slightly up to 5 for extremely.

Correlation coefficient analyses between PANAS and 5-item Non-burnout Inventory negative affect and positive affect scores were also performed. The Non-burnout Inventory positive affect score was the sum of the enthusiastic and energetic scores. The PANAS positive affect score was the sum of the alert, enthusiastic, interested, excited, strong, proud, inspired, attentive, active, and determined scores. The Non-burnout Inventory negative affect score was the sum of the overwhelmed, people demanding, and drained scores. The PANAS negative affect score was the sum of the irritated, nervous, distressed, upset, scared, guilty, hostile, ashamed, jittery, and afraid scores. The PANAS and Non-burnout Inventory positive affect items were coded as 1 for very slightly up to 5 for extremely. The PANAS and Non-burnout Inventory negative affect items were coded as 1 for very slightly up to 5 for extremely.

### Concurrent validity of the Maslach

A correlation analysis was conducted between the Non-burnout Inventory positive affect and negative affect scores and the 3 Maslach domains. The Non-burnout Inventory positive affect score was the sum of the energetic and enthusiastic scores, coded as 1 for very slightly or not at all up to 5 for extremely. The Non-burnout Inventory negative affect score was the sum of the overwhelmed, drained, and people too demanding scores, coded as 1 for very slightly or not at all up to 5 for extremely. The Maslach personal accomplishment score was the sum of the effective, positive influence, and exhilarated scores. The Maslach emotional exhaustion score was the sum of the drained, fatigued, and strained scores. The Maslach depersonalization score was the sum of the objects, callous, and do not care scores. All Maslach items were coded as 0 for never up to 6 for every day.

A second correlation analysis was conducted to assess the relationships between the Non-burnout Inventory total score and Maslach items. The Non-burnout Inventory positive affect score was the sum of the energetic and enthusiastic scores, coded as 1 for very slightly or not at all up to 5 for extremely. The Non-burnout Inventory negative affect score was the sum of the overwhelmed, drained, and people too demanding scores, coded as 5 for very slightly or not at all to 1 for extremely. The Non-burnout Inventory total score was the sum of the Non-burnout Inventory positive affect and negative affect scores. The Maslach personal accomplishment score was the sum of the effective, positive influence, and exhilarated scores, coded as 0 for every day up to 6 for never. The Maslach emotional exhaustion score was the sum of the drained, fatigued, and strained scores, coded as 0 for never up to 6 for every day. The Maslach depersonalization score was the sum of the objects, callous, and do not care scores, coded as 0 for never up to 6 for every day. The Maslach total score was the sum of the personal accomplishment, emotional exhaustion, and depersonalization scores.

### Statistical analyses

Results were entered into an Excel 2010 worksheet (Microsoft Corp., Redmond, WA, USA) and imported into the SAS System for Windows, release 9.2 (SAS Institute Inc., Cary, NC, USA). All mean values were accompanied by their standard deviation. SAS was used to perform the CALIS procedure (PROC CALIS), using the maximum likelihood least squares estimation, and the factor procedure (PROC FACTOR) for confirmatory and exploratory factor analyses, respectively. Concurrent validity analyses were assessed in SAS using Spearman Rank-order correlation procedures where the level of significance was *p* < 0.05.

## Results

### Wellbeing inventory survey responses

Of the 419 participants who completed the Wellbeing Inventory, the negative affect items rated moderately to extremely were as follows: irritation, 145 (34.6%); nervousness, 113 (27.0%); overreaction, 69 (16.5%); tension, 182 (43.4%); overwhelmed, 159 (38.0%); people demanding, 126 (30.0%); and drained, 146 (34.8%). The numbers of participants with negative affect items rated moderately to extremely were as follows: 138 (32.9%), 0 of 7 items; 281 (67.1%) ≥1 of 7 items; 229 (54.7%), ≥2 of 7 items; and 167 (39.9%), ≥3 of 7 items. The positive affect items rated very slightly or none at all to moderately were as follows: restful sleep, 324 (77.3%); energetic, 317 (75.7%); alert, 239 (57.0%); and enthusiastic, 308 (73.5%). The numbers of participants with positive affect items rated very slightly or none at all to moderately were as follows: 30 (7.2%), 0 of 4 items; 389 (92.8%), ≥1 of 4 items; 336 (80.2%), ≥2 of 4 items; and 287 (68.5%), ≥3 of 4 items.

### PANAS and Maslach survey responses

For the 191 participants who completed the PANAS, the mean positive affect score was 31.9 ± 7.2 and the mean negative affect score was 17.6 ± 5.3. Overall, 150 participants completed the Maslach survey. The mean scores for the Maslach were as follows: emotional exhaustion, 9.4 ± 4.2; depersonalization, 4.9 ± 3.8; and personal accomplishment, 13.9 ± 3.0.

### Reliability and factor analyses of the wellbeing inventory

The Cronbach alpha coefficient was assessed from 114 Wellbeing Inventory surveys completed by the 2018 BIS-neurofeedback participants on learning days 1 and 2 of the study. The Cronbach alpha coefficients were 0.7604 for the 4 positive affect variables and 0.8306 for the 7 negative affect variables. The Varimax rotation exploratory factor analysis loadings are shown in Table 1 and the analysis demonstrated that factor 1 and factor 2 item loadings are prominent and distinct.

**Table 1:** Wellbeing Inventory exploratory factor loadings (Varimax rotation)

	Factor 1	Factor 2
	(NA Items)	(PA Items)
Restful Sleep	0.1420	0.4656
Energetic	0.1277	0.8903
Alert	0.0176	0.7721
Enthusiastic	0.0556	0.8073
Irritated	0.7408	−0.0597
Nervous	0.6250	−0.0696
Overreaction	0.5941	0.1715
Tension	0.8357	0.1076
Overwhelmed	0.7789	0.1148
People Demanding	0.5531	0.1999
Drained	0.7321	0.3007

Wellbeing Inventory: St. Elizabeth Youngstown Hospital Wellbeing Inventory; *NA* negative affect, *PA* positive affect

The initial exploratory factor analysis potentially identified a 3-factor model: 2 eigenvalues were compelling (4.76 and 1.81) and 1 eigenvalue was marginal (1.05). For the 3-factor model, the cumulative eigenvalue was 0.66 and the total communality was 7.1. The 3-factor model created appropriate loading of the 4 positive affect items on a single factor; however, the 7 negative affect items variably loaded on the other 2 factors. Of greatest concern was that 4 of the 7 negative affect items competitively loaded on both of the 2 negative affect factors. That is, these 4 negative affect items had factor loadings ≥0.4 on both of the 2 negative affect factors. We then explored a 2-factor model that showed that the cumulative eigenvalue was 0.57 and the total communality was 6.2. For the 2-factor model, the inter-factor correlation was 0.31. We selected the 2-factor model, because the factor loadings were persuasive, the cumulative eigenvalue was 86.4% of the 3-factor model (0.57 ÷ 0.66), the total communality was 87.3% of the 3-factor model (6.2 ÷ 7.1), the inter-factor correlation was 0.31, and this model paralleled the PANAS paradigm. For the 2-factor model, the mean 11-item communality was 0.56 ± 0.15 (range 0.3 to 0.8; proportion ≥ 0.4 90.9% [10/11]; proportion ≥ 0.6 63.6% [7/11]).

Overall, 419 participant surveys were utilized to conduct the confirmatory factor analysis for the Wellbeing Inventory. The fit indices were as follows: root mean square residual, 0.07; standardized root mean square residual, 0.07; goodness of fit index, 0.93; root mean square error of approximation estimate, 0.09; root mean square error of approximation estimate upper 95% confidence interval, 0.10; Bentler Comparative Fit Index, 0.92; Bentler-Bonett Non-normed Index (Tucker Lewis Index), 0.90; and absolute fit chi-square *p*-value, < 0.0001.

### Reliability and factor analyses of the non-burnout inventory

Analysis of the 419 Non-burnout Inventory surveys showed that the alpha coefficients were 0.7642 for the 2 positive affect variables and 0.7859 for the 3 negative affect variables. Factor 1 and factor 2 item loadings are shown in Table 2, and were found to be prominent and distinct. The initial exploratory factor analysis identified a 2-factor model: both eigenvalues were compelling (2.95 and 1.36) and all other eigenvalues were much lower than 1.0. The cumulative eigenvalue was 0.74 and the total communality was 3.7. The inter-factor correlation was 0.36. The mean 5-item communality was 0.75 ± 0.08 (range 0.6 to 0.8; proportion ≥ 0.6100% [5/5]).

**Table 2:** Non-burnout Inventory exploratory factor loadings (Varimax rotation)

	Factor 1	Factor 2
	(NA Items)	(PA Items)
Energetic	0.1223	0.8885
Enthusiastic	0.0843	0.8956
Overwhelmed	0.8228	0.0882
People Demanding	0.7945	0.0332
Drained	0.8709	0.1852

Non-burnout Inventory: St. Elizabeth Youngstown Hospital Non-burnout Inventory; *NA* negative affect, *PA* positive affect

Using the 419 participant surveys, the confirmatory factor analysis fit indices were as follows: root mean square residual, 0.02; standardized root mean square residual, 0.02; goodness of fit index, 0.99; root mean square error of approximation estimate, 0.07; Bentler Comparative Fit Index, 0.99; Bentler-Bonett Non-normed Index (Tucker Lewis Index), 0.97; and absolute fit chi-square *p*-value, 0.0259.

### Concurrent validity of the PANAS

In total, 191concomitant Wellbeing Inventory and PANAS negative affect and positive affect scores were computed. The Wellbeing Inventory positive affect and PANAS positive affect scores had a significant correlation (*r* = 0.85; *p* < 0.0001). The Wellbeing Inventory negative affect and PANAS negative affect scores also had a significant correlation (*r* = 0.88; *p* < 0.0001).

Non-burnout Inventory and PANAS negative affect and positive affect scores were computed from the 191concomitant Wellbeing Inventory and PANAS surveys. The Non-burnout Inventory positive affect and PANAS positive affect scores had a significant correlation (*r* = 0.87; *p* < 0.0001). The Non-burnout Inventory negative affect and PANAS negative affect scores also had a significant correlation (*r* = 0.74; *p* < 0.0001).

### Concurrent validity of the Maslach

Overall, 150 concomitant Non-burnout Inventory and Maslach surveys were collected. The correlations between Non-burnout Inventory positive affect and negative affect scores and Maslach scores are shown in Table 3. The Non-burnout Inventory total score had significant relationships with the Maslach total score (*r* = − 0.56; *p* < 0.0001), emotional exhaustion (*r* = − 0.68; *p* < 0.0001), personal accomplishment (*r* = − 0.20; *p* = 0.0143), and depersonalization (*r* = − 0.28; *p* = 0.0004).

**Table 3:** Non-burnout Inventory PA and NA score correlations with the Maslach scores

Non-burnout Inventory Score	Maslach Score	r-value	*p*-value
PA	Emotional Exhaustion	−0.48	< 0.0001
NA	Emotional Exhaustion	0.64	< 0.0001
PA	Personal Accomplishment	0.29	0.0003
NA	Depersonalization	0.27	0.0008
NA	Callous	0.36	< 0.0001

Non-burnout Inventory: St. Elizabeth Youngstown Hospital Non-burnout Inventory; *PA* positive affect, *NA* negative affect, *Maslach* Maslach Burnout Inventory

## Discussion

Internal reliability, exploratory and confirmatory factor analyses, and concurrent validity assessments indicate that the Wellbeing Inventory and Non-burnout Inventory have acceptable psychometric performance. In addition, our study findings verified our aforementioned hypotheses.

### Survey responses

Of concern, but expected, are some of the Wellbeing Inventory negative affect item responses. Tension, feeling overwhelmed, feeling that people were too demanding, and feelings of being drained were each rated moderately to extremely in 30–40% of participants. Of further note is the fact that ≥2 of 7 negative affect items were rated moderately to extremely in half of the participants, and ≥ 3 items were found in 40% of the participants. The PANAS negative affect mean score was essentially the same as that found in an investigation of 150 Canadian physicians, suggesting that the current survey findings are likely representative of other physician/nurse providers [16]. The PANAS positive affect mean score is also quite similar to that found in the same investigation, further suggesting that the current survey findings are likely representative of other physician/nurse providers [16]. The mean scores for the 3 abbreviated Maslach domains are similar to those found in other United States healthcare practitioners, findings suggesting that healthcare burnout is a consistent and concerning problem [13].

### Reliability and validity of the wellbeing inventory

The Cronbach alpha coefficient for the 2-factor Wellbeing Inventory was relatively good. The exploratory factor analysis results showed that factor 1 and factor 2 item loadings were prominent and distinct. Further, the confirmatory factor analysis fit indices indicated that the model was acceptable. Importantly, the Wellbeing Inventory positive affect and PANAS positive affect scores had a strong association and the Wellbeing Inventory negative affect score had a strong association with the PANAS negative affect score. Based on these findings, the Wellbeing Inventory has internal consistency, and it is a valid indicator of wellbeing, relative to positive affect and negative affect assessments.

### Adequacy of the sample size

The initial sample size target was to obtain at least 20 participants for each item (*n* = 220). This number was provided by the BIS neurofeedback study (*n* = 228). In order to assess correlations between our Wellbeing Inventory and PANAS, we captured Wellbeing Inventory item responses for an additional 191 participants to yield a total of 419 surveys. We thought that the sample size was adequate, because 1) there were at least 20 participants for each survey item; 2) the Cronbach alpha was acceptable, 3) the exploratory factor loadings were compelling; 4) confirmatory factor analysis was acceptable; and 5) concurrent criterion validity correlations were significant. Based on these findings, we determined that this provided a reasonable appraisal of the Wellbeing Inventory. Further evidence that the sample size is adequate relates to the ratio of the number of items relative to the number of factors and to the communality values of the 11-item Wellbeing Inventory and the 5-item Non-burnout Inventory [17]. Certainly, obtaining similar psychometric properties in a larger cohort would be more compelling.

### Reliability and validity of the non-burnout inventory

The Cronbach alpha coefficient for the 2-factor Non-burnout Inventory was relatively good. Results of the exploratory factor analysis showed that factor 1 and factor 2 item loadings were large and discriminating. Additionally, the confirmatory factor fit criteria were quite compelling. Of importance, the Non-burnout Inventory positive affect and PANAS positive affect scores had a strong association, as did the Non-burnout Inventory negative affect and PANAS negative affect scores. Further, the Non-burnout Inventory positive affect and negative affect scores had moderate and strong associations, respectively, with Maslach emotional exhaustion. The Non-burnout Inventory positive affect and negative affect scores had significant associations with Maslach personal accomplishment and depersonalization domains. Denollet and De Vries have shown that significant associations between PANAS positive affect and negative affect and the 3 Maslach domains exist in other populations [18].

The Non-burnout Inventory total score had a good or moderate association with the Maslach total score and a strong correlation with the Maslach emotional exhaustion domain. The total Non-burnout Inventory score was coded such that a high score would likely suggest that burnout might be relatively low, that is, positive affect is relatively high and negative affect is relatively low. Whereas, the Maslach total score ratings were constructed to suggest that a high score would suggest that emotional exhaustion and depersonalization would be relatively frequent and personal accomplishment would be relatively infrequent. Of relevance, Durak et al. have shown that the total Maslach score has a significant association with PANAS positive affect and negative affect scores [19].

### Study limitations

The principal limitation of the current investigation is the failure to assess predictive validity or concomitant behavioral appraisals. A second limitation is that the focus on physicians and nurses fails to assess the relevance of the survey to non-physician/nurse healthcare workers or other hospital employees. To mitigate potential privacy concerns, we did not include epidemiologic details of the participants; however, this might have limited the identification of factors that could have correlated with adverse experiential results. The sample size is relatively small.

## Conclusions

According to the current survey results and germane literature, positive affect, negative affect, and burnout profiles suggest that physician and nurse provider experiential limitations are relatively common. The Wellbeing Inventory was found to be reliable, demonstrate latent construct validity, and reveal concomitant correlations with another standard survey. Likewise, the Non-burnout Inventory was also shown to be internally consistent, possess structural validity, and document concurrent associations with other recognized wellbeing and burnout survey tools. Because the sample size is relatively small, ascertaining similar psychometric properties in a larger cohort would be more compelling. We believe that the 11-item Wellbeing Inventory is shorter and less redundant than the PANAS, requires less contemplation than the Maslach, and represents a measure for assessing positive affect, negative affect, and burnout. The St. Elizabeth Youngstown Hospital instruments should be considered by other investigators as reasonable methodologies for monitoring experiential perceptions in physician and nurse care providers.

## Data Availability

The datasets generated during and/or analyzed during the current study are not publicly available due to statutory provisions regarding data and privacy protection, but are available from the corresponding author on reasonable request.

## Abbreviations

BIS: Bispectral Index™
Maslach: Maslach Burnout Inventory
Non-burnout Inventory: St. Elizabeth Youngstown Hospital Non-burnout Inventory
PANAS: Positive and Negative Affect Schedule
Wellbeing Inventory: St. Elizabeth Youngstown Hospital Wellbeing Inventory
